# Targeting Multiple Tumors Using T-Cells Engineered to Express a Natural Cytotoxicity Receptor 2-Based Chimeric Receptor

**DOI:** 10.3389/fimmu.2017.01212

**Published:** 2017-09-29

**Authors:** Vasyl Eisenberg, Katerina Shamalov, Shimrit Meir, Shiran Hoogi, Rhitajit Sarkar, Shirel Pinker, Gal Markel, Angel Porgador, Cyrille J. Cohen

**Affiliations:** ^1^The Laboratory of Tumor Immunology and Immunotherapy, The Mina and Everard Goodman Faculty of Life Sciences, Bar-Ilan University, Ramat Gan, Israel; ^2^Faculty of Health Sciences, The Shraga Segal Department of Microbiology, Immunology and Genetics, The National Institute for Biotechnology in the Negev, Ben-Gurion University of the Negev, Beer Sheva, Israel; ^3^ASAS, Amity University Haryana, Manesar, India; ^4^The Ella Lemelbaum Institute of Immuno-Oncology, Institute of Oncology, Sheba Medical Center, Tel Hashomer, Israel

**Keywords:** NCR2, T-cells engineering, chimeric receptors, adoptive T-cell transfer, T-cell immunotherapy, natural killer cells

## Abstract

Recent developments in cancer treatment are demonstrating the increasing and powerful potential of immunotherapeutic strategies. In this regard, the adoptive transfer of tumor-specific T-lymphocytes approaches can lead to tumor regression in cancer patients. More recently, the use of T-cells genetically engineered to express cancer-specific receptors such as the anti-CD19 chimeric antigen receptor (CAR) continues to show promise for the treatment of hematological malignancies. Still, there is a crucial need to develop efficient CAR-T cell approaches for the treatment of solid tumors. It has been shown that other lymphocytes such as natural killer (NK) cells can demonstrate potent antitumor function—nonetheless, their use in immunotherapy is rather limited due to difficulties in expanding these cells to therapeutically relevant numbers and to suppression by endogenous inhibitory mechanisms. Cancer recognition by NK cells is partly mediated by molecules termed natural cytotoxicity receptors (NCRs). In the present study, we hypothesize that it is possible to endow T-cells with an NK recognition pattern, providing them with a mean to recognize tumor cells, in a non-MHC restricted way. To test this, we genetically modified human T-cells with different chimeric receptors based on the human NCR2 molecule and then assessed their antitumor activity *in vitro* and *in vivo*. Our results show that expression in primary lymphocytes of an NCR2-derived CAR, termed s4428z, confers T-cells with the ability to specifically recognize heterogeneous tumors and to mediate tumor cytotoxicity in a mouse model. This study demonstrates the benefit of combining tumor recognition capability of NK cells with T cell effectiveness to improve cancer immunotherapy.

## Introduction

Recent advances in tumor immunotherapy have demonstrated a crucial role for immunosurveillance, as tumor development and progression can be often linked to a failure of the immune system to recognize tumors and to mount an adequate response (1–3). In that regard, natural killer (NK) cells represent a central component of the early anticancer immune response. The latter relies on the integrated balance of signals transduced by activating and inhibitory receptors (4–6). Known activating receptors are DNAM-1, NKG2D, and members of the natural cytotoxicity receptors (NCRs) family: NCR1 (NKp46), NCR2 (NKp44), and NCR3 (NKp30) (7). NCR2/Nkp44 was identified by Vitale et al. two decades ago (8) and several studies described its role as an activating receptor important for the function of NK cells (9). NCR2 is involved in tumor recognition and can mediate cytokine and cytotoxic granule secretion (10, 11), but some of its isoforms (such as Nkp44-1) bear an ITIM and can dampen NK cell function (11). The ligands of NCRs are often found on viral-infected and tumor cells and as such, NCR molecules can mediate the recognition of a wide range of tumors and their lysis by NK cells (11–20). NCR ligands have also the potential to regulate the receptor function and facilitate tumor escape mechanisms from NK cells (11, 21, 22), which would lend support to their wide expression on the surface of cancer cells. In particular, newly discovered NCR2 ligands such as PCNA (11, 21) and MLL5/NKp44L (23) were showed to be broadly expressed by several tumors and recognized by NK-cells. It would, therefore, be valuable to target them in the context of antitumor immunotherapy and targeted therapies.

Compared to T-cell-based immunotherapy, NK-based treatment of cancer is less prevalent (6), since it has often proven difficult to expand and adequately condition these lymphocytes (24), which may display poor reactivity *in vivo* (25) and may be rejected when administered in allogeneic settings (26, 27). On the other hand, adoptive T-cell transfer can mediate the regression of large solid and hematological malignancies (28–31). In addition to the use of naturally occurring tumor-specific T-cells, we and others showed the feasibility of engineering lymphocytes to express T-cell receptors (TCRs) conferring them novel antitumor activity (32). However, it is important to remember that TCR-based therapy is dependent on the expression of a specific HLA allele that present the targeted epitope (33), thereby restricting its applicability to selected patients. Another type of MHC-independent T-cell specificity engineering approach can be achieved by chimeric antigen receptors (CARs) targeting tumor-surface antigens. CARs are composed of a targeting portion (generally a scFv specific for a defined antigen) and a signaling moiety (incorporating parts of FcRIIIγ or CD3ζ molecules) (28, 34). Still, these approaches are often specific for a defined antigen, whose expression may be limited to certain types of cancer, mainly hematological. Importantly, the use of T-cells virally transduced with an anti-CD19 CAR can lead to up to 80–90% complete regression in ALL patients as well as unprecedented therapeutic efficacy in the treatment of other hematological malignancies (30, 35, 36). Nonetheless, there is a crucial need to develop efficient CAR-T cell approaches for the treatment of solid tumors (37). To extend the use of chimeric molecules using receptors derived from another type of lymphocytes (NK cells), we and others recently showed that NCR-1 and NCR-3 can be used a targeting moieties in a novel type of CARs derived from NK-cell receptors (38, 39) and that these molecules can mediate the recognition of diverse cancers and eradicate tumors *in vivo*. In the present manuscript, we address the case of NCR2 (also known as NKp44) and show that an optimal NCR2/CD28z chimeric receptor, termed s4428z, mediates antitumor response against multiple tumors *in vitro*, which translated into high cytokine secretion, superior expression of activation markers, and cytotoxicity *in vitro* and *in vivo* using a mouse model of human xenograft tumors.

## Materials and Methods

### PBMCs and Cell Lines

PBLs used in this study were from normal donors from the Israeli Blood Bank (Tel-Hashomer, Israel) after obtaining an informed consent. MDA-MB-435 (ATCC/HTB-129) is a previously described malignant breast line derived from metastatic site (pleural effusion) but now considered a melanoma line, DU-145 (ATCC/HTB-81) is a prostate cancer line derived from metastatic site (brain), HeLa (ATCC/CCL-2) is a cervix adenocarcinoma line, and Colo-205 (ATCC/CCL-222) is a colorectal adenocarcinoma cell line derived from metastatic site (ascites). Generation of primary melanoma cultures, M#3 and M#14, was performed as part of clinical adoptive transfer protocols, which were approved by the Israel Ministry of Health (Approval no. 3518/2004, ClinicalTrails.gov Identifier NCT00287131), after obtaining an informed consent from the patients. Packaging line 293GP (expressing GAG and POL) was described (40). Tumor cells were cultured in RPMI (Invitrogen, Carlsbad, CA, USA) or in DMEM (Invitrogen, Carlsbad, CA, USA), both supplemented with 10% FBS (Biological Industries, Beth Haemek, Israel). Lymphocytes were cultured in BioTarget medium (Biological Industries, Beth Haemek, Israel), 10% FBS, and 300 IU/ml IL-2. Cells were maintained at 37°C and 5% CO_2_.

### NCR2 Chimeras and Retroviral Constructs

**Figure 1:**
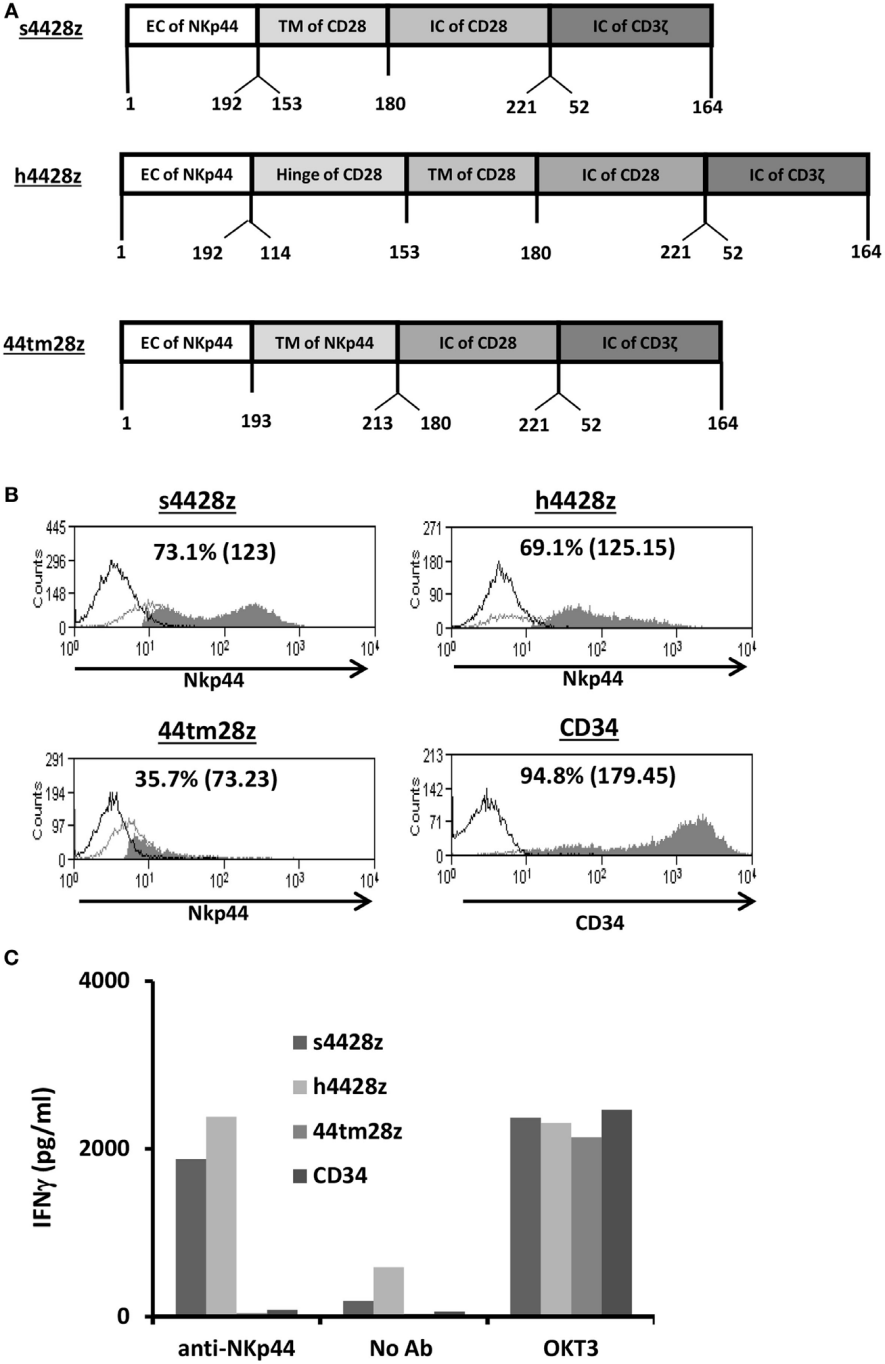
Design and expression of NCR2-based chimeras. **(A)** Schematic representation of the different NCR-2-based chimeric receptors. The amino acid numbering (based on the original protein) is indicated below each segment. **(B)** OKT3-stimulated human primary PBLs were transduced with the different versions of NCR2-based chimeric antigen receptor or with truncated CD34 (control gene) as indicated. Transgene expression was assessed by flow cytometry. The dotted line represents the staining of the mock-transduced control. The percentage of positive cells and the MFI (in brackets) are shown. These results are representative of six independent experiments with at least four different donors and the difference between the population transduced with NCR2-chimeric molecules and the control population was found statistically significant (*p* < 0.05; calculated using a Student’s paired *t*-test). **(C)** These cells (10^5^) were incubated in a 96-well plate in the presence of plate-bound anti-NCR2 (0.2 μg/well) or OKT3 for 16 h. IFNγ secreted in the coculture supernatant was measured by ELISA.

The cDNA encoding the human NKp44 (NCR2) was amplified from reverse-transcribed mRNA isolated from human NK cells. The different chimeras were created by overlapping PCR (41, 42) and their amino acid composition is indicated in Figure 1A. These chimeras as well as a truncated version of CD34 were cloned into the well-characterized retroviral vector backbone pMSGV1 (38), which is a derivative of the murine stem cell virus (MSCV)-based splice-gag vector (pMSGV), and which uses a MSCV long terminal repeat.

### Transduction of PBLs

For virus production, transfection of 2 × 10^6^ 293GP cells with 9 µg DNA of MSGV1-based retroviral construct and 4.5 µg envelop plasmid (VSV-G) was performed using JetPrime transfection reagent (Polyplus, France) (43–45). Retroviral supernatant was collected 36 h after the DNA transfection. Freshly isolated human PBLs were stimulated for 48 h in the presence of 50 ng/ml OKT3 (eBioscience, San Diego, CA, USA) before transduction. Following stimulation, lymphocytes were transduced with retroviral vectors by transfer to non-treated tissue culture dishes (Nunc, Rochester, NY, USA) that had been precoated with RetroNectin (Takara, Japan) and retroviral vectors as previously described (43).

### FACS Analysis and Antibodies

Fluorophore-labeled anti-human CD8, CD137, CD25, and CD34 were purchased from BioLegend (San Diego, CA, USA). To stain for human NCR2, we used the 3.43.13 mAb (generously provided by Dr. Marco Colonna, Washington University, St. Louis, MO, USA). Immunofluorescence, analyzed as the relative log fluorescence of live cells, was measured using a CyAn-ADP flow cytometer (Beckman Coulter, Brea, CA, USA). Approximately 1 × 10^4^–1 × 10^5^ cells were analyzed. Cells were stained in a FACS buffer made of PBS, 0.5% BSA, and 0.02% sodium azide.

### Cytokine Release Assays

Lymphocyte cultures were tested for reactivity in cytokine release assays using commercially available ELISA kits for IL-2, IFNγ, and TNFα (R&D Systems, Minneapolis, MN, USA). For these assays, 1 × 10^5^ responder cells (T-cells) and 1 × 10^5^ stimulator cells (tumor cells) were incubated in a 0.2-ml culture volume in individual wells of 96-well plates. Stimulator cells and responder cells were cocultured for 18 h. Cytokine secretion was measured in culture supernatants diluted to be in the linear range of the assay. As a control for T cell activity, we incubated the different T-cell cultures with PMA/ionomycin at a concentration of 50 ng/ml and 1 µM, respectively.

### Cell Separation

T-cell populations were separated using a magnetic bead-based approach for negative selection (EasySep TM—StemCell Technologies Inc., Canada).

### Cell-Mediated Cytotoxicity Assay

Target cells were labeled with 2 µM CFSE (eBioscience, San Diego, CA, USA) for 6 min and then cocultured with transduced lymphocytes at 37°C for 4 h, at different effector:target (E:T) ratios (as indicated in figure legend). After the coculture, propidium iodide (PI) 1 µM (Sigma-Aldrich, Israel) was added for assigning the ratio of cell death. Samples were analyzed by flow cytometry.

### *In Vivo* Cytotoxicity Assay—Winn Assay

6 to 8 weeks year-old NOD-SCD-IL2R-mice (Har-lan, Jerusalem, Israel) were inoculated in the flank with a mixture of 2 × 10^6^ HeLa cells or MDA-MB-435 and 2 × 10^6^ transduced lymphocytes resuspended in 40 μl Biotarget medium. Tumor size was measured every 3 days using a caliper in a blinded fashion. This study was carried out in accordance with the recommendations of the Bar-Ilan university committee for animal welfare and the Israel Ministry of Health. The protocol was approved by the Bar-Ilan university committee for animal welfare.

### Statistical Analysis

Cytokine secretion, marker upregulation, and cytotoxic activity were compared using a paired Student’s *t*-test. Tumor growth slopes were compared using one-way ANOVA. *p* < 0.05 was considered statistically significant.

## Results

### Evaluation of Different NCR2-Based Chimeric Receptors

We constructed three different NCR2-chimeric receptors by fusing the extracellular domain of NCR2 (NKp44) to either the transmembrane domain of CD28 (s4428z) or to that of NCR2 (44tm28z) or the hinge of CD28 (h4428z), all of which connected to the CD28 and CD3ζ signaling domains (Figure 1A). These were cloned into the MSGV1 retroviral vector and were transduced into OKT3-stimulated primary human PBLs. 48 h after transduction, we determined the surface expression levels of the different receptors by flow cytometry. We show in Figure 1B that we could detect expression of all the transduced receptors, with s4428z and h4428z demonstrating high expression levels with 73.1% (MFI = 123) and 69.1% (MFI = 125), respectively (control gene truncated CD34 showed 94.8% positive cells). These levels of expression were constant for 30 days following transduction without any selection and the growth and expansion rates of the NCR2-CAR transduced population were comparable to those of the CD34 control T-cells (data not shown).

We then tested the basic function of these receptors by incubating these cells in the presence of plate-bound anti-NCR2 (as a positive control for T-cell activation, we used OKT3 antibody). After 16 h, we harvested the supernatant and measured IFNγ concentrations by ELISA. We found that s4428z and h4428z mediated the highest secretion of IFNγ compared to 44tm28z or the CD34 control (1,876 and 2,393 vs. 40 and 80 pg/ml, respectively). These results correlate with the expression data presented in Figure 1B and suggest that NCR2-based chimeras can function in human T-cells.

### s4428z Mediates the Efficient Recognition of Tumors of Different Histologies

NCR2 ligands are expressed by tumors (11, 21, 23) and can be recognized by NK cells, though often dampening the function of the latter as we previously showed (21). We decided to test the function of T-cells equipped with the NCR2-CARs described in Figure 1 and to examine to what extent these chimeric receptors would mediate antitumor activity. In parallel, we assessed the expression levels of NCR2-ligands by staining different tumor cell lines with a NCR2/NKp44-Fc molecule and detected significant ligand expression on several tumor lines (MDA-MB-435, HeLa, and DU-145; Figure S1 in Supplementary Material) while the colon carcinoma line Colo205 showed no significant staining.

**Figure 2:**
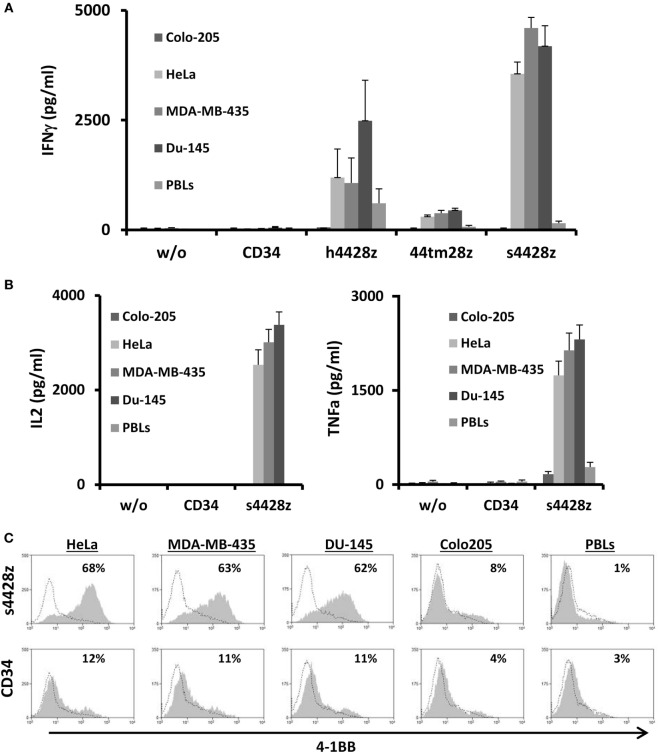
Antitumor function of the NCR2-based chimeras. **(A)** Human primary lymphocytes were transduced with a retroviral vector encoding either h4428z, s4428z, 44tm28z, and truncated CD34 (control). Transduced T cells were cocultured with different tumor lines or normal PBLs as indicated. IFNγ secreted in the coculture supernatant was measured by ELISA. These results are presented as mean + SEM (*n* = 5, with three different donors). **(B)** s4428z- or CD34-transduced T-cells were cocultured with the indicated target cells and IL2 (right panel) and TNFα (left panel) concentration secreted in the culture supernatant was determined by ELISA. These results are presented as mean + SEM (*n* = 5, with three different donors). **(C)** Transduced PBLs with either s4428z or CD34 cells were cocultured with tumor lines as indicated and analyzed by flow cytometry for 4-1BB expression gated on the CD8^+^ population. The percentage of positive cells is shown and the dotted line represents the marker staining of the control. These results are representative of three independent experiments with different donors and the difference between s4428z and CD34 was found to be statistically significant (*p* < 0.05, calculated using a Student’s paired *t*-test).

We assessed the antitumor function mediated by the different NCR2-CARs by setting up a coculture of s4428z-, h4428z-, 44tm28z-, and CD34-transduced primary human T-cells with the aforementioned tumor lines. We detected specific cytokine secretion mediated by the NCR2-chimeras with s4428z demonstrating the highest of all compared to the CD34-transduced control group (e.g., 4,597 vs. 51 pg/ml of IFNγ in coculture with the cancer line MDA-MB-435; *p* < 0.05—Figure 2A). We did not notice any significant cytokine secretion in coculture with CD34 transduced cells or in the presence of Colo205 or normal PBLs (negative controls).

Based on the results presented in Figure 2A, we selected s4428z as our optimal NCR2-based CAR and examined if this receptor could facilitate the secretion of additional cytokines important for T-cell function and antitumor activity such as IL2 and TNFα (46). As seen in Figure 2B, significant amounts of both aforementioned cytokines were secreted by T-cells expressing s4428z (e.g., 3,381 pg/ml of IL2 and 2,310 pg/ml of TNFα in cocultures with DU145).

We then decided to examine if NCR2-chimeric receptor may cause the upregulation of a T-cell activation marker. s4428z- or truncated CD34-transduced T-cells were cocultured overnight with several cancer cell lines and were analyzed for surface expression of the activation marker 4-1BB (CD137). We observed that s4428z-engineered CD8^+^ cells significantly upregulated the surface expression of 4-1BB compared to control T-cells (Figure 2C—e.g., 68% of 4-1BB-positive cells compared to 12% for control CD34^+^). No significant staining was observed in cocultures with the negative control cell line Colo205 or normal PBLs.

**Figure 3:**
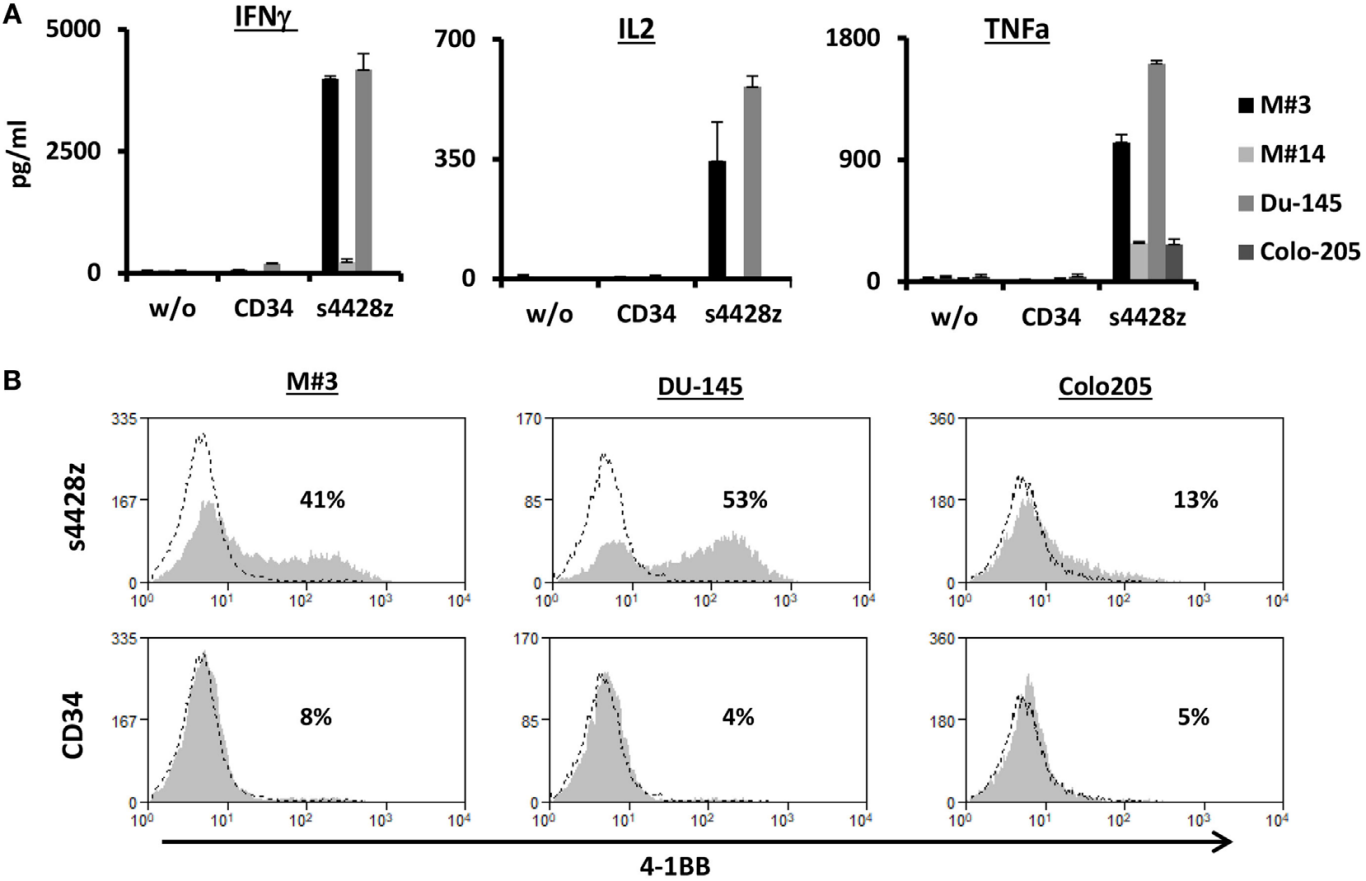
Function of the s4428z chimera against cells derived from primary melanoma culture. **(A)** Human primary lymphocytes were transduced with a retroviral vector encoding s4428z and truncated CD34 (control). Transduced T cells were cocultured with cells derived from primary melanoma cultures (M#3 and M#14) as well as two control tumor lines (DU145 positive and Colo205 negative controls). IFNγ, IL2, and TNFα secreted in the coculture supernatant was measured by ELISA. These results are presented as mean + SEM (*n* = 3, with two different donors). **(B)** Transduced PBLs with either s4428z or CD34 cells were cocultured with the indicated targets and analyzed by flow cytometry for 4-1BB expression gated on the CD8^+^ population. The percentage of positive cells is shown and the dotted line represents the marker staining of the control. These results are representative of three independent experiments with different donors and the difference between s4428z and CD34 was found to be statistically significant (*p* < 0.05, calculated using a Student’s paired *t*-test).

Additionally, we assessed whether the s4428z chimeric receptor could mediate the recognition of cells from primary tumor culture. To that end, we used two targets derived from primary melanoma cultures, namely M#3 and M#14. As controls, we used the DU145 and Colo205 cell lines. As seen in Figure 3A, s4428z-equipped T-cells secreted significant amounts of IFNγ, IL2, and TNFα when cocultured with cells from the primary melanoma culture M#3 (e.g., 3,987, 343, and 1,027 pg/ml, respectively), underscoring the potential clinical relevance of our approach. Moreover, we observed an increased surface expression of the T-cell activation marker 4-1BB by s4428z-expressing T-cells cocultured with M#3 (Figure 3B—e.g., 41% of 4-1BB-positive cells compared to 8% for control CD34^+^ transduced T-cells). Overall, these results indicate that s4428z can mediate the efficient recognition of tumors of multiple histologies.

### s4428z Chimeric Receptor Can Facilitate the Activation CD4^+^ T-Lymphocytes

**Figure 4:**
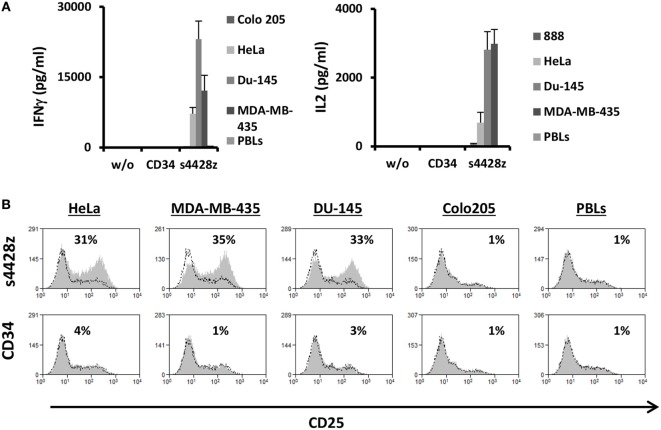
s4428z function in CD4^+^ cells. **(A)** s4428z- or CD34-transduced purified CD4^+^ cells were cocultured with different tumor lines or not as indicated. IFNγ (right panel) and IL2 (left panel) secreted in the coculture supernatant was measured by ELISA. These results are representative of three independent experiments, performed with different donors and the difference between the s4428z and CD34 populations was found statistically significant (*p* < 0.05, calculated using a Student’s paired *t*-test). **(B)** CD4^+^-transduced T cells were analyzed by flow cytometry for CD25 expression following overnight coculture with target cells as indicated. The percentage of positive cells is shown and the dotted line represents the marker staining of the control. These results are representative of three independent experiments and the difference between S4428z and CD34 was found to be statistically significant (*p* < 0.05, calculated using a Student’s paired *t*-test).

As widely reported, CD4^+^ T-cell responses are important to the coordination of immune responses (47–49). In contrast to TCRs, CARs (including the aforementioned s4428z chimeric receptor) may function in a non-MHC-restricted manner. Therefore, we surmised that the latter should exert an immunostimulatory effect on CD4^+^ T cells. To test this, we purified human primary CD4^+^ cells and transduced them with either s4428z or truncated CD34. We cocultured these cells with different cancer lines and analyzed IL-2 and IFNγ secretion as well as activation marker upregulation. As expected, s4428z mediated a significant cytokine secretion by transduced lymphocytes compared to the CD34 control (e.g., 23,078 pg/ml of IFNγ and 2,818 pg/ml of IL2 in cocultures with the DU145 target cell line—Figure 4A). Moreover, s4428z caused a specific upregulation of the activation marker CD25 in CD4^+^ T-cells as seen in Figure 4B; e.g., 34.6% of CD25^+^ cells compared to 1% for the CD34 control in coculture with the MDA-MB-435 cell line. These results show that s4428z can provide CD4^+^ T-lymphocytes with direct antitumor functionality.

### s4428z Mediates Cytotoxicity *In Vitro* and *In Vivo*

**Figure 5:**
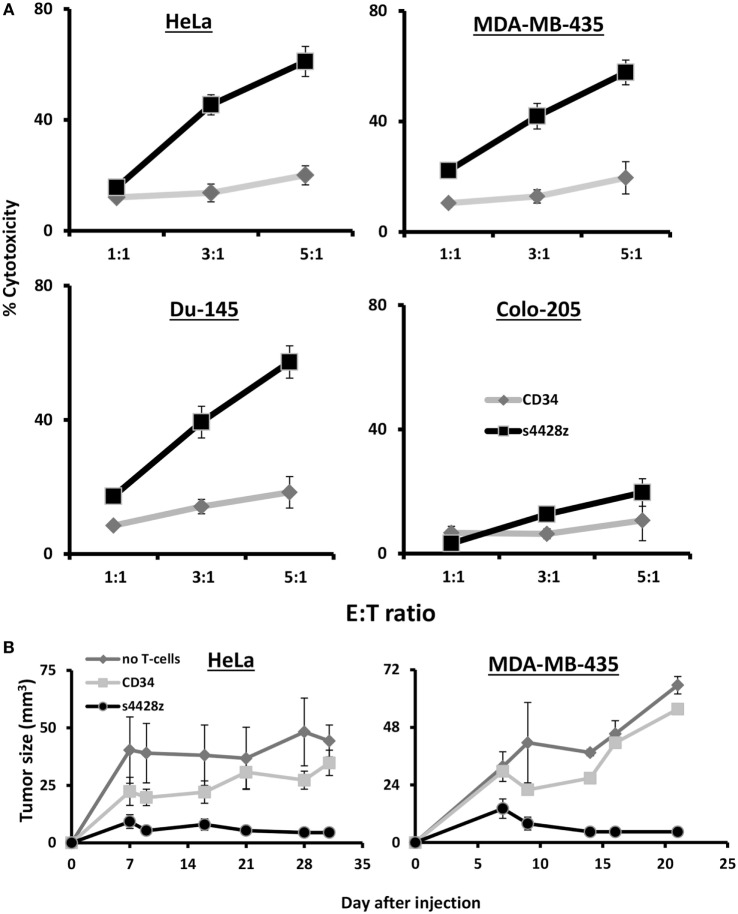
s4428z mediates antitumor cytotoxic activity. **(A)** s4428z- or CD34-transduced cells were cocultured with the indicated CFSE-labeled tumor cells at the indicated (E:T) ratios. After 4 h, propidium iodide was added and the cells were analyzed by flow cytometry. Cytotoxicity was calculated based on the CFSE+/PI+ population out of the total CFSE+ population. These results are presented as mean + SEM of three independent experiments with three different donors and the difference between the s4428z and CD34 populations was found statistically significant (*p* < 0.05, calculated using a Student’s paired *t*-test). **(B)** Winn assay. NS-G mice were inoculated with HeLa or MDA-MB-435 cells and transduced lymphocytes (either s4428z, CD34—control or not as indicated) in the flank. Tumor growth was measured in a blinded fashion using a caliper and calculated using the following formula: (*D* × *d*^2^) × Π6, where *D* is the largest tumor diameter and *d* its perpendicular one. These results are representative of two experiments (four to five mice in the treated groups) and are shown for the different time points as mean + SEM. The difference between the s4428z and CD34-treated groups was found statistically significant (*p* < 0.05).

We decided to examine the potential of s4428z in mediating T-cell cytotoxic activity and set up cell-mediated cytotoxicity assays; we labeled tumor cells with CFSE and cocultured those with CD8^+^ T-cells transduced to express the s4428z construct. We analyzed cytotoxicity by flow cytometry after adding to those cocultures PI, which stained dead cells and we observed a statistically significant antitumor cytotoxic activity mediated by s4428z-transduced lymphocytes as shown by the extent of PI-positive population (Figure 5A—58% for s4428z vs. 18% for CD34-transuced cells using the DU145 target cell-line at E:T 5:1; *p* < 0.05).

Finally, the antitumor cytotoxic activity of s4428z-transduced T-cells was further confirmed *in vivo* assays*;* we tested the ability of s4428z-transduced T-cells to mediate enhanced tumor killing activity in Winn assays using a xenograft model of human tumor. NS-G mice were inoculated with a mixture of either HeLa or MDA-MB-435 cells and s4428z- or CD34-transduced T lymphocytes (at E:T of 1:1), and we measured tumor development in the following weeks. We show in Figure 5B that the s4428z-treated group displayed a statistically significant reduced tumor growth when compared to the CD34 or untreated (control) group for both tumor targets (*p* < 0.05). We thus conclude that the s4428z chimeric receptor is capable of mediating antitumor cytotoxicity both in *in vitro* and *in vivo* settings.

## Discussion

Immunotherapy treatments have achieved widespread clinical benefits, proving that the immune system is capable of containing and eradicating tumors. In that regard, the use of CAR therapy is becoming a promising strategy for the treatment of cancer (28), mainly applied to hematological malignancies. Herein, in order to develop novel CARs targeting solid malignancies, we explored the possibility of utilizing a member of the natural cytotoxicity receptor family, NCR2, as a targeting moiety to redirect T-lymphocytes specificity toward a panel of solid tumors, taking advantage of NCR2-ligand expression by cancer cells.

We designed three NCR2-based chimeric receptors and evaluated their function in primary human T-cells. We mainly modified either the hinge or transmembrane domains as these can considerably influence CAR function (44). Indeed, as seen in Figure 1B, the NCR2-based CAR incorporating the original hinge/transmembrane region of NCR2 showed a decreased stability, which apparently led to poor function (Figure 1C). It appears, therefore, that CD28 transmembrane region is important for NCR2-CAR function. Additionally, our results with the different NCR2/CD28 CARs studied herein emphasize the need to evaluate both the expression and the function of chimeric receptors empirically as previously recommended (42, 50, 51). Also, since several studies showed that the inclusion of other co-stimulatory domains [derived for example from CD27 (52), 41BB (53–55), OX40 (56)] may result in additional CAR functionality, further optimization is warranted.

Following basic characterization, s4428z was selected as our optimal construct and was expressed in human primary T-cells to enable the targeting of tumors of multiple histologies. An added value to this approach would be the possibility to target several malignancies using the same non-MHC restricted receptor (38, 39, 57–60). Such a CAR could, therefore, represent a valuable and available reagent for the immunotherapeutic treatment of numerous cancer patients. Unlike in the case of NK cells in which NCR2-ligands can induce immunosuppressive signals (11), s4428z-expressing T-cells can derive benefit from high NCR2-ligand expression. Yet, since the exhaustive identification of NCR2 cellular ligands is still an ongoing task (7), one should consider including a suicide gene in the therapeutic vector configuration when envisaging clinical applications (61). Nonetheless, our *in vivo* assays (Figure 5B) provide preliminary and promising preclinical data exemplifying the therapeutic potential of the present approach.

We show in Figure 4 that s4428z can mediate antitumor function when expressed in CD4^+^ human primary T-lymphocytes. The option of recruiting CD4^+^ T lymphocytes in the microenvironment is attractive as they may provide some local support to sustain the CTL antitumor response (62–64). Nevertheless, such option should be ultimately applied only to certain helper T-cell populations such as Th1 but not Th17 or Tregs as these bear an uncertain role in tumor promotion *via* inflammation or may dampen antitumor immunity, respectively. It has been suggested that NCR expression may promote the rapid response to pathogens in a non-antigen-specific manner (65), and we and others have shown that NCR2 is important to the recognition and elimination of certain bacterial and viral infections (19, 66). Therefore, the extension of NCR-based CAR to target the treatment of such infections should be evaluated.

In conclusion, we have designed in the present study an optimized NCR2-based CAR capable of targeting multiple malignancies. This study demonstrates the benefit of combining tumor recognition capability of NK cells with T cell effectiveness to improve cancer immunotherapy, and we trust the kind of approach can improve the versatility of treatments based on the adoptive transfer of genetically engineered T-cells.

## Ethics Statement

This study was carried out in accordance with the recommendations of the Bar-Ilan university committee for animal welfare and the Israel Ministry of Health. The protocol was approved by the Bar-Ilan university committee for animal welfare. Generation of primary melanoma cultures, M#3 and M#14, was performed as part of clinical adoptive transfer protocols, which were approved by the Israel Ministry of Health (Approval no. 3518/2004, ClinicalTrails.gov Identifier NCT00287131), after obtaining an informed consent from the patients.

## Author Contributions

VE performed the experiments, analyzed the data, wrote the manuscript, and prepared the figures. KS, SM, SH, and RS performed experiments and analyzed data. SP performed experiments. GM provided vital reagents and designed experiments. AP analyzed data. CC designed experiments, analyzed data, and wrote the manuscript.

## Conflict of Interest Statement

The authors declare that the research was conducted in the absence of any commercial or financial relationships that could be construed as a potential conflict of interest.
