# Supplementary figures and images for: Targeting Multiple Tumors Using T-Cells Engineered to Express a Natural Cytotoxicity Receptor 2-Based Chimeric Receptor
Source: Front Immunol. 2017 Sep 29;8:1212. doi: 10.3389/fimmu.2017.01212 (PMC5649149; doi:10.3389/fimmu.2017.01212)

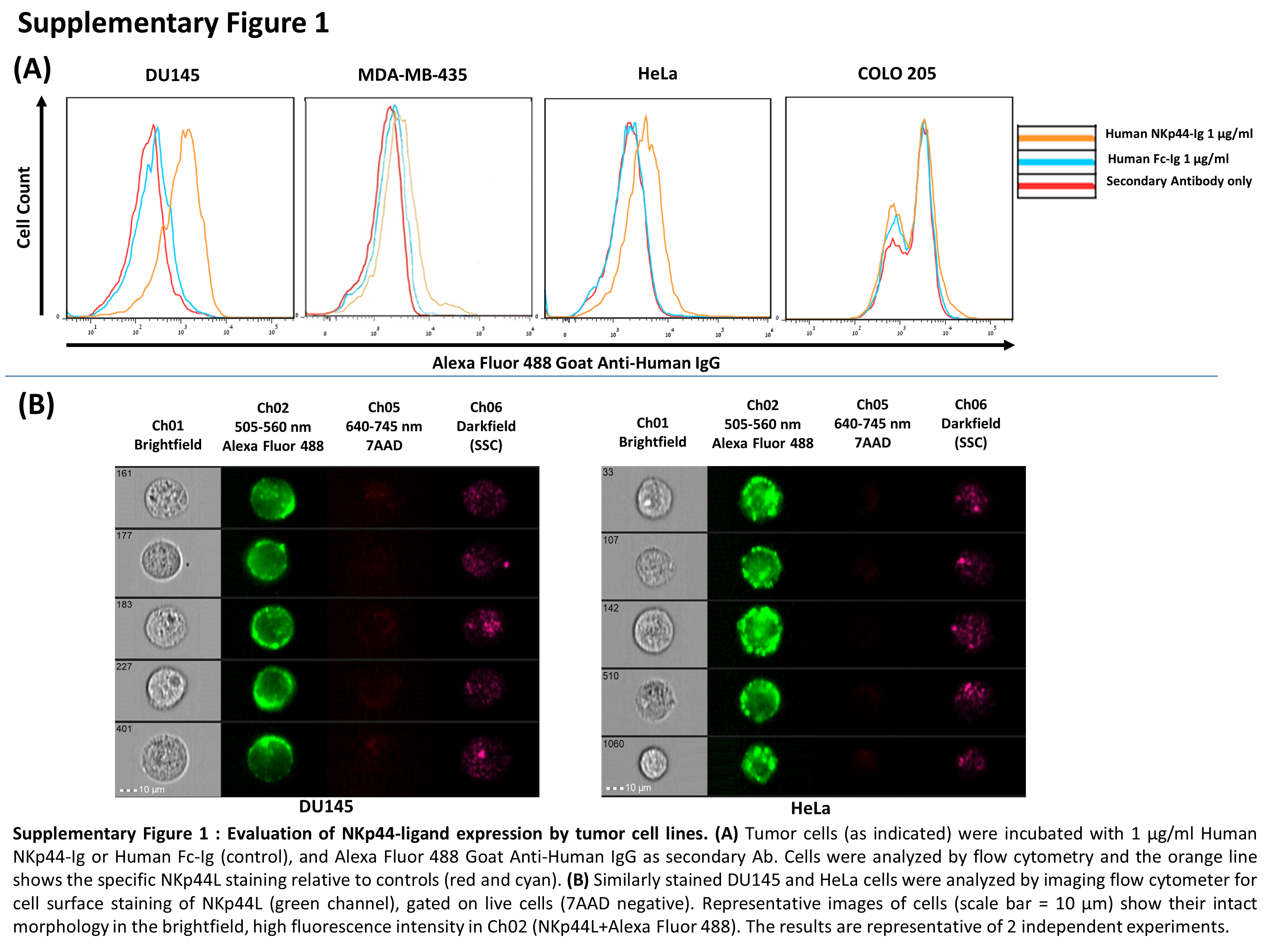

Supplement: Supplementary file 1 [file Image_1.TIF]
